# A Novel Approach to the Management of Dentin Dysplasia Using Zygoma Implants: A Case Report

**DOI:** 10.7759/cureus.68099

**Published:** 2024-08-29

**Authors:** Gauri Sharma, Bhushan Mundada, Nitin Bhola, Rozina Vishnani, Deepankar Shukla, Ankita Pathak

**Affiliations:** 1 Oral and Maxillofacial Surgery, Sharad Pawar Dental College and Hospital, Datta Meghe Institute of Higher Education and Research, Wardha, IND; 2 Prosthodontics, Sharad Pawar Dental College and Hospital, Datta Meghe Institute of Higher Education and Research, Wardha, IND

**Keywords:** atypical dentin, implant, rootless teeth, permanent dentition, dentin dysplasia

## Abstract

Dentin dysplasia (DD) is a rare clinical entity that can affect deciduous dentition alone, or both deciduous and permanent dentition. It is a developmental disorder that can be classified as DD type I or type II. This case report describes a rare case of DD type I in a 19-year-old patient, highlighting the clinical presentation and the radiographic features of the condition, confirmed by ground sectioning and microscopic examination of extracted teeth. The case report also describes the management of this patient using zygoma and endosteal implants.

## Introduction

Chamberlain was the first person to describe the absence of root formation in teeth and coined the term "rootless teeth" [1]. Dentin dysplasia (DD) is a hereditary developmental anomaly of defective dentin formation. It is further subclassified into DD type I and type II [2]. The type I variant of DD typically affects root formation, with a normal-appearing crown. DD type II has similar clinical features to dentinogenesis imperfecta. In contrast to the type I variant, root formation is unaffected in DD type II [2,3]. The simultaneous presence of both subtypes has also been reported in the literature [4]. The defective root formation in DD type I causes mobility of teeth and may lead to premature exfoliation. In this case report, we describe the case of a 19-year-old male patient who presented with the above condition at our Outpatient Department (OPD).

## Case presentation

**Figure 1:**
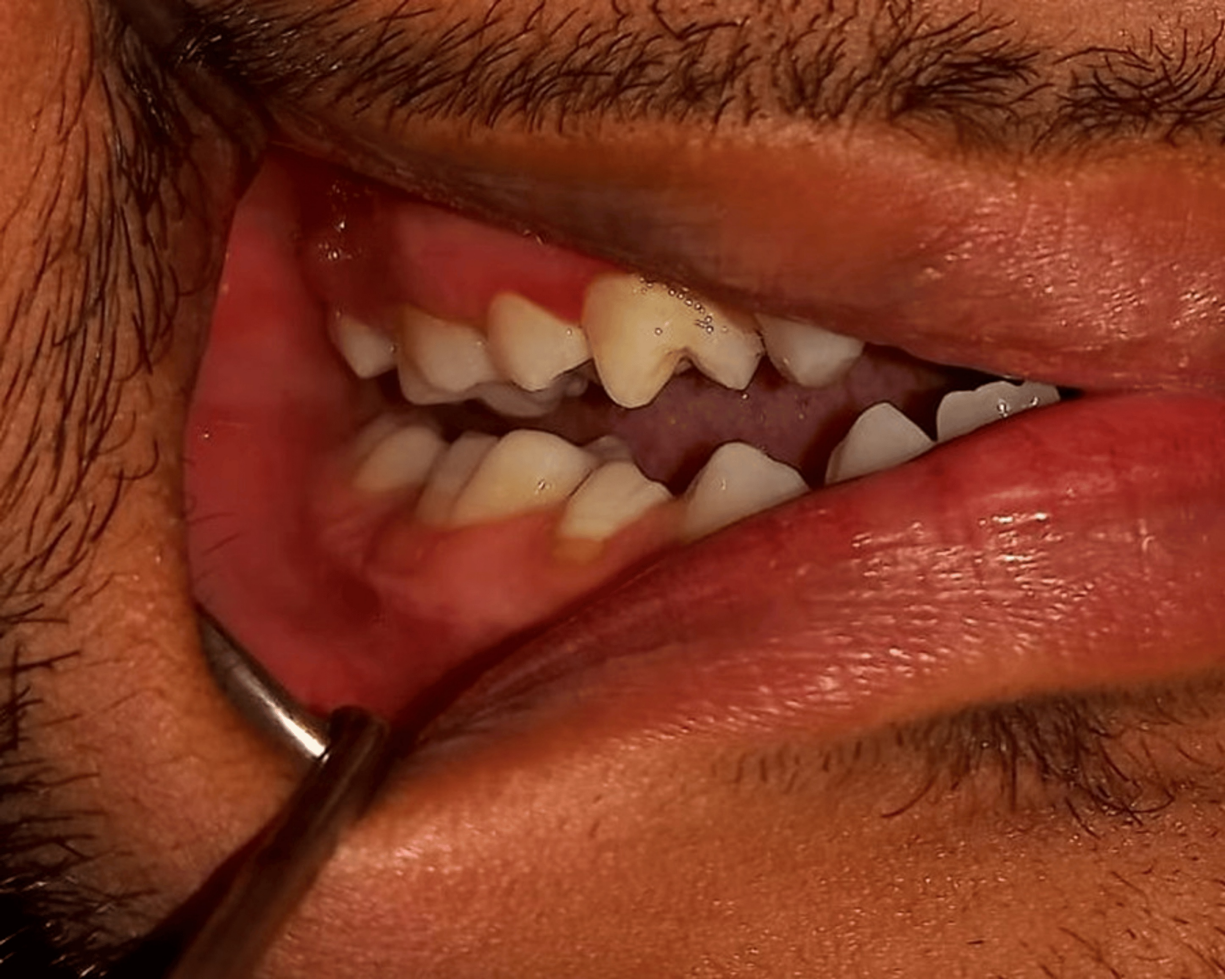
Right-sided preoperative occlusion of the patient Image credit: Dr. Gauri Sharma

**Figure 2:**
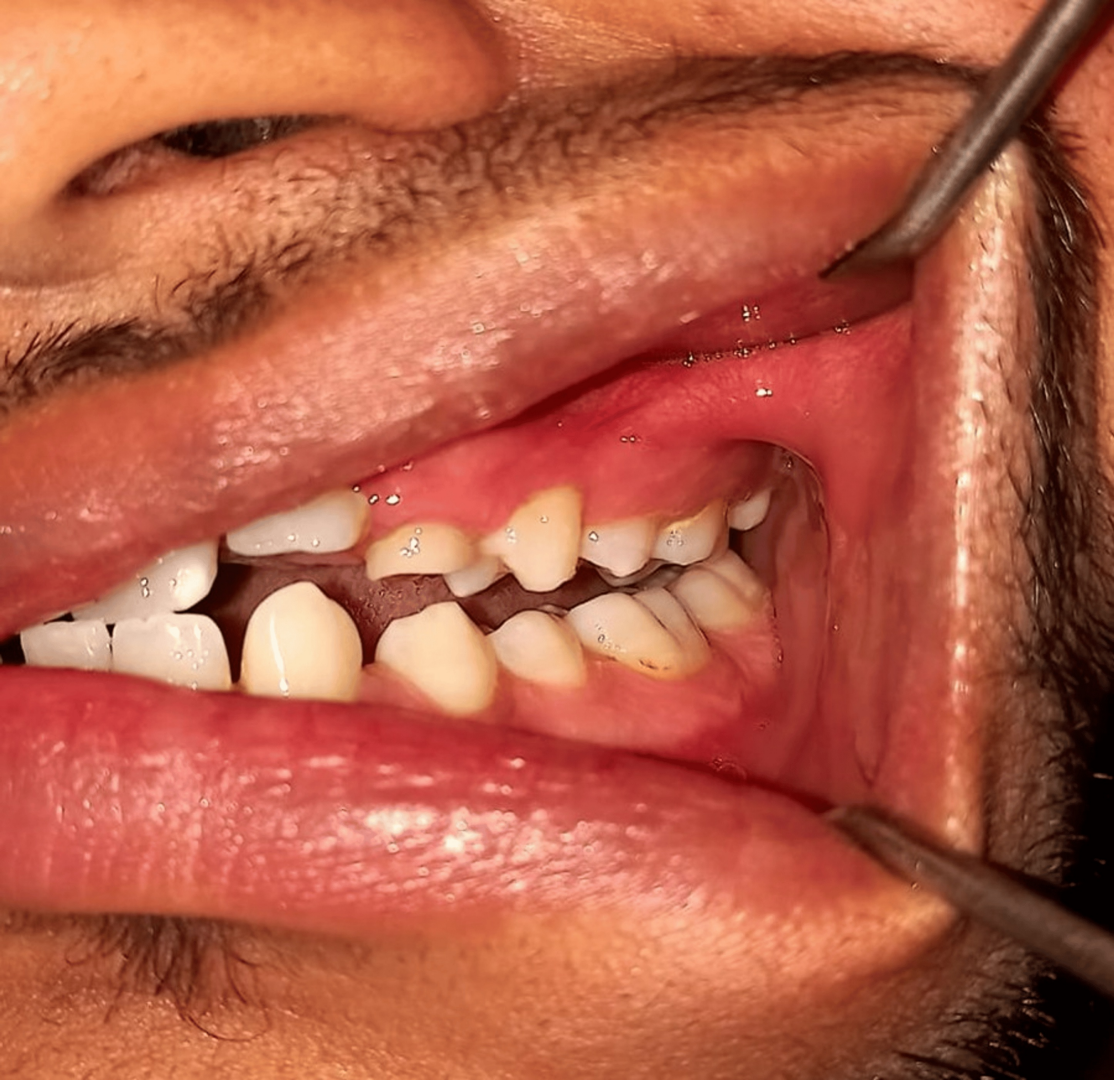
Left-sided preoperative occlusion of the patient Image credit: Dr. Gauri Sharma

**Figure 3:**
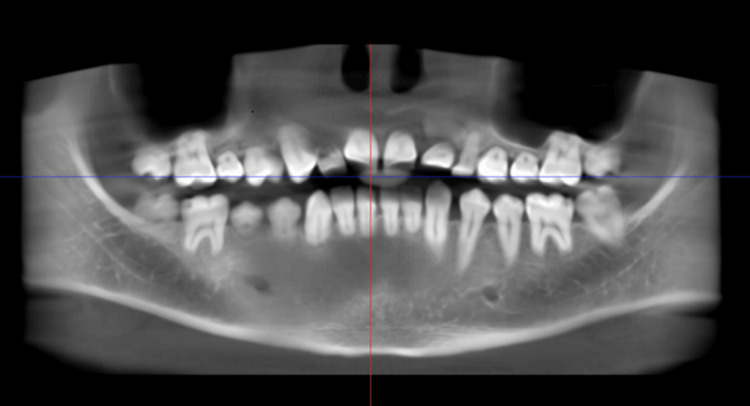
CBCT of the patient showing generalized shortening of roots with normal crowns, which is consistent with dentin dysplasia Image credit: Dr. Gauri Sharma CBCT: Cone-beam computed tomography

A 19-year-old male patient reported to the Oral and Maxillofacial Surgery OPD with a complaint of generalized teeth mobility. The patient did not provide any relevant medical or family history. After examining the patient, a provisional diagnosis of juvenile periodontitis was made. However, upon evaluating the orthopantomogram (OPG), a rudimentary or complete absence of root formation was noted in all the teeth, with no evidence of bone loss. Hence, a developmental disorder was suspected as the cause of the rootless teeth. Based on these findings, a diagnosis of DD was made. To rule out the possibility of any associated syndrome, the patient was advised to undergo serum alkaline phosphatase and calcium testing, as well as a full-mouth cone-beam computed tomography (CBCT). The patient’s blood investigation reports were within normal limits (Figures 1-3).

The surgical team advised the patient to undergo the extraction of all teeth, followed by rehabilitation via an implant-supported prosthesis. The treatment plan consisted of placing two pterygoid/zygoma implants, three to four endosteal implants in the maxillary arch, and eight endosteal implants in the mandibular arch, with delayed loading.

**Figure 4:**
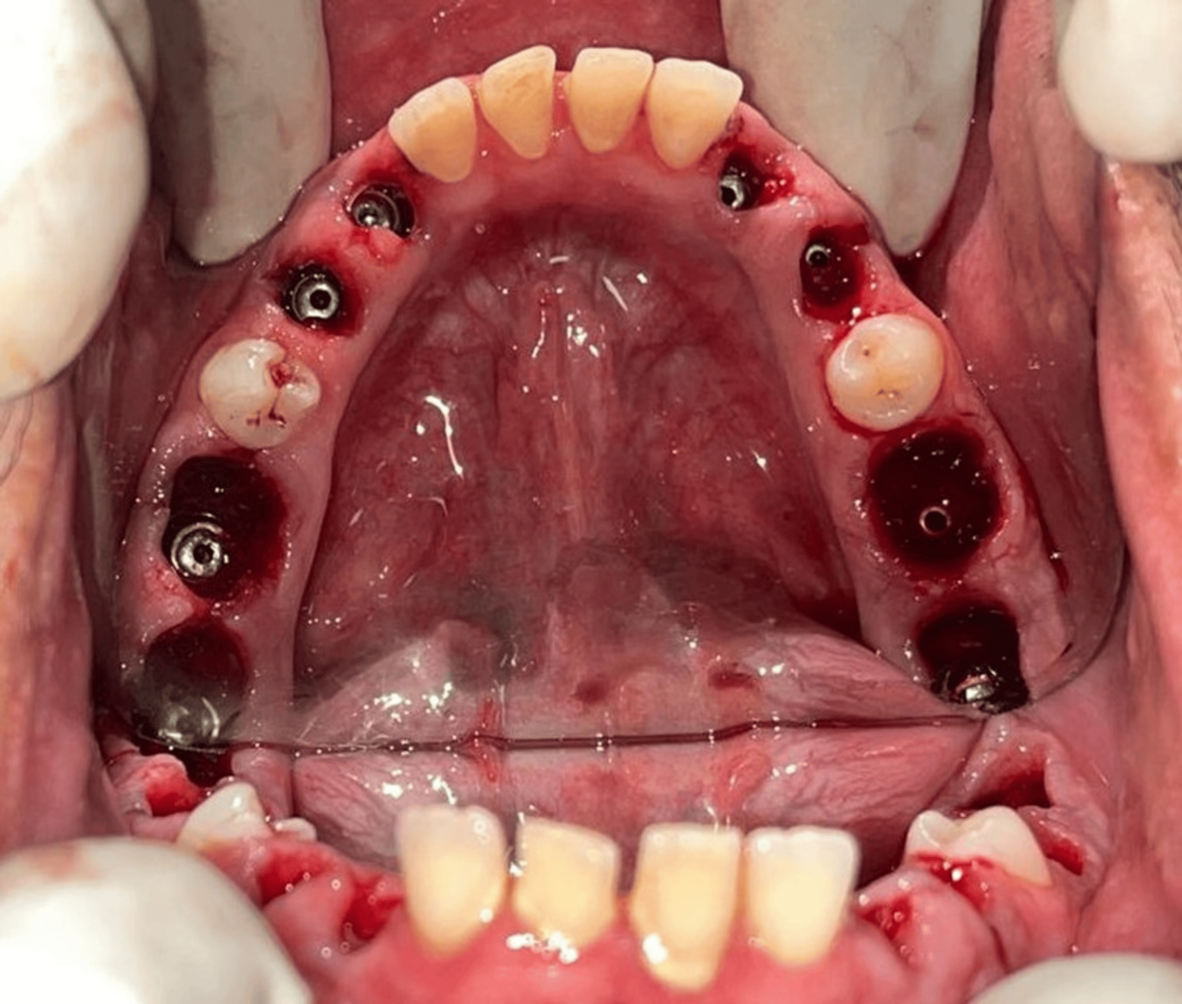
Intraoperative view of eight endosteal implants placed in the mandibular arch All the mandibular teeth were extracted after the implant placements. Image credit: Dr. Ankita Pathak

The placement of eight immediate mandibular implants was done first, and the surgery was uneventful. The patient was recalled after five days for the placement of implants in the maxillary arch (Figure 4).

**Figure 5:**
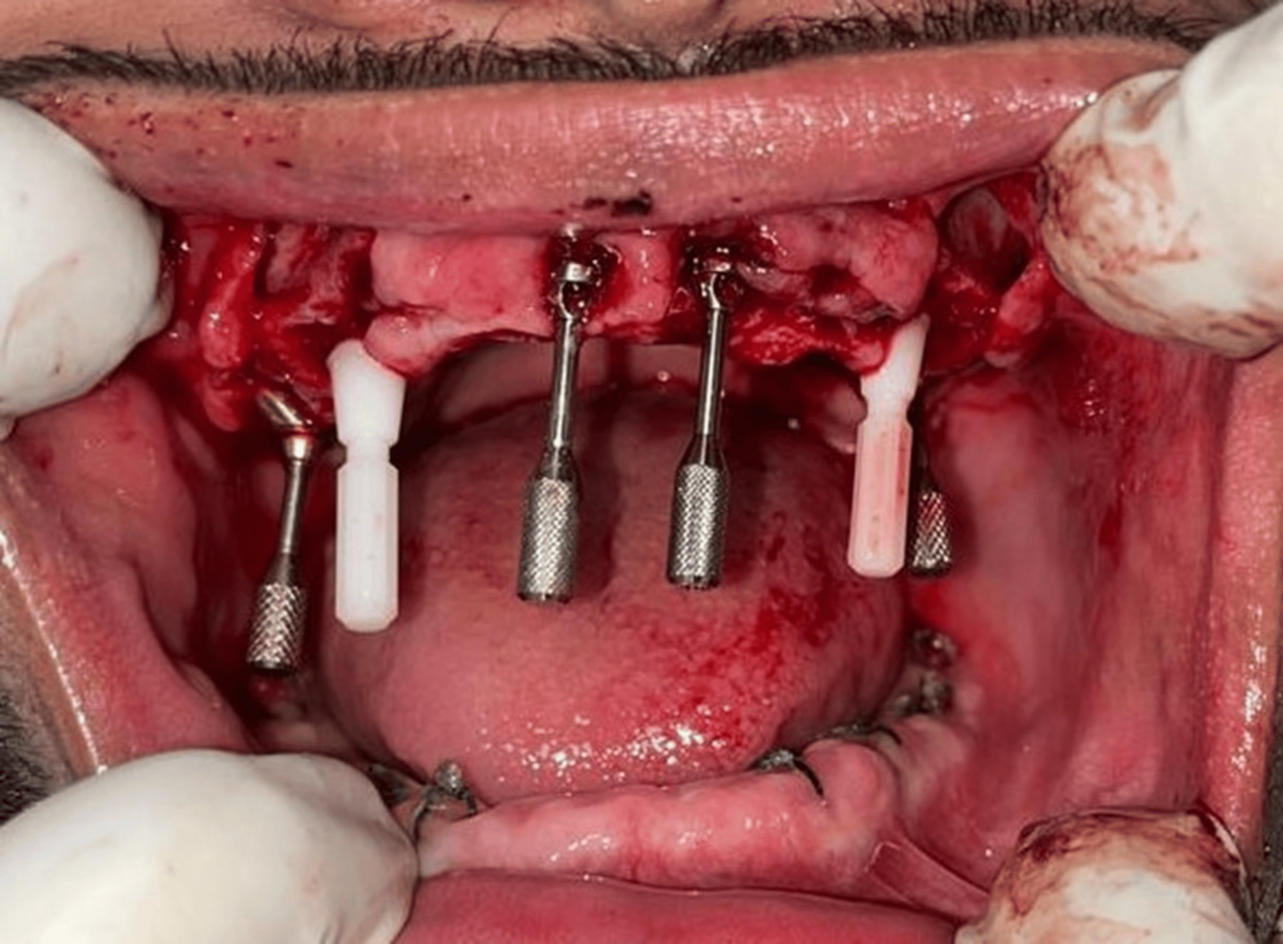
Intraoperative view of the maxillary arch of the patient showing two zygoma implants and four endosteal implants Image credit: Dr. Ankita Pathak

**Figure 6:**
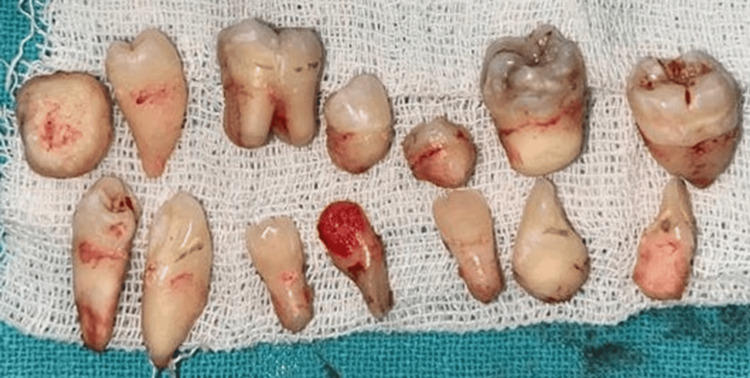
Extracted teeth of the patient showing the abnormal anatomical pattern of the roots Image credit: Dr. Ankita Pathak

The implant surgery for the upper arch was initiated by placing a pterygoid implant in the right quadrant. However, the implant failed to engage with adequate torque. Therefore, the decision was made to use zygoma implants instead of pterygoid implants. Two zygoma implants, along with four endosteal implants in the canine-premolar region, were placed. The patient was recalled after seven days for suture removal and review. Meanwhile, the patient was prescribed calcium and vitamin D3 tablets once a week for 12 weeks (Figures 5-6).

**Figure 7:**
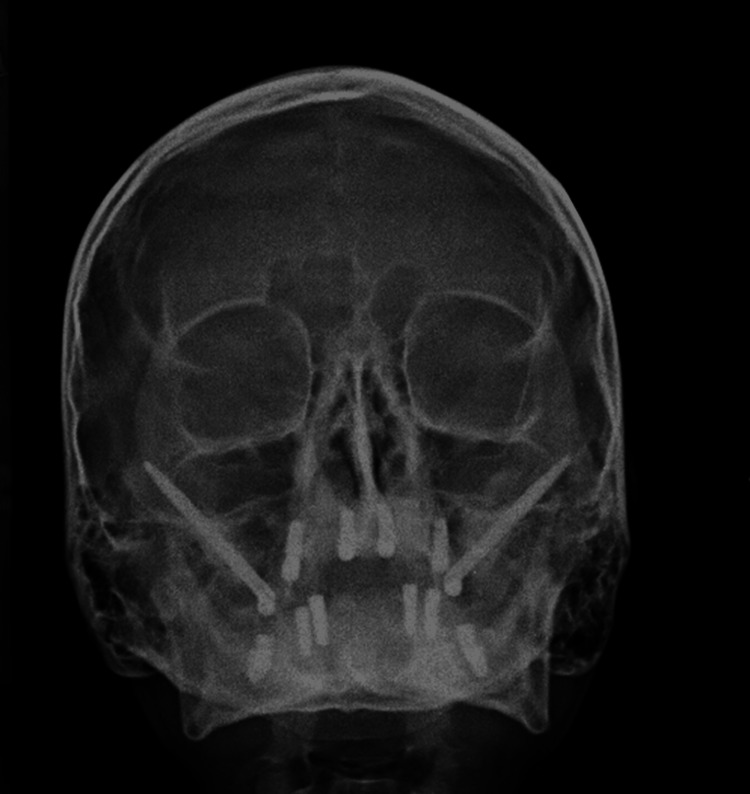
Postoperative PNS X-ray of the patient Image credit: Dr. Bhushan Mundada PNS: Paranasal sinus

**Figure 8:**
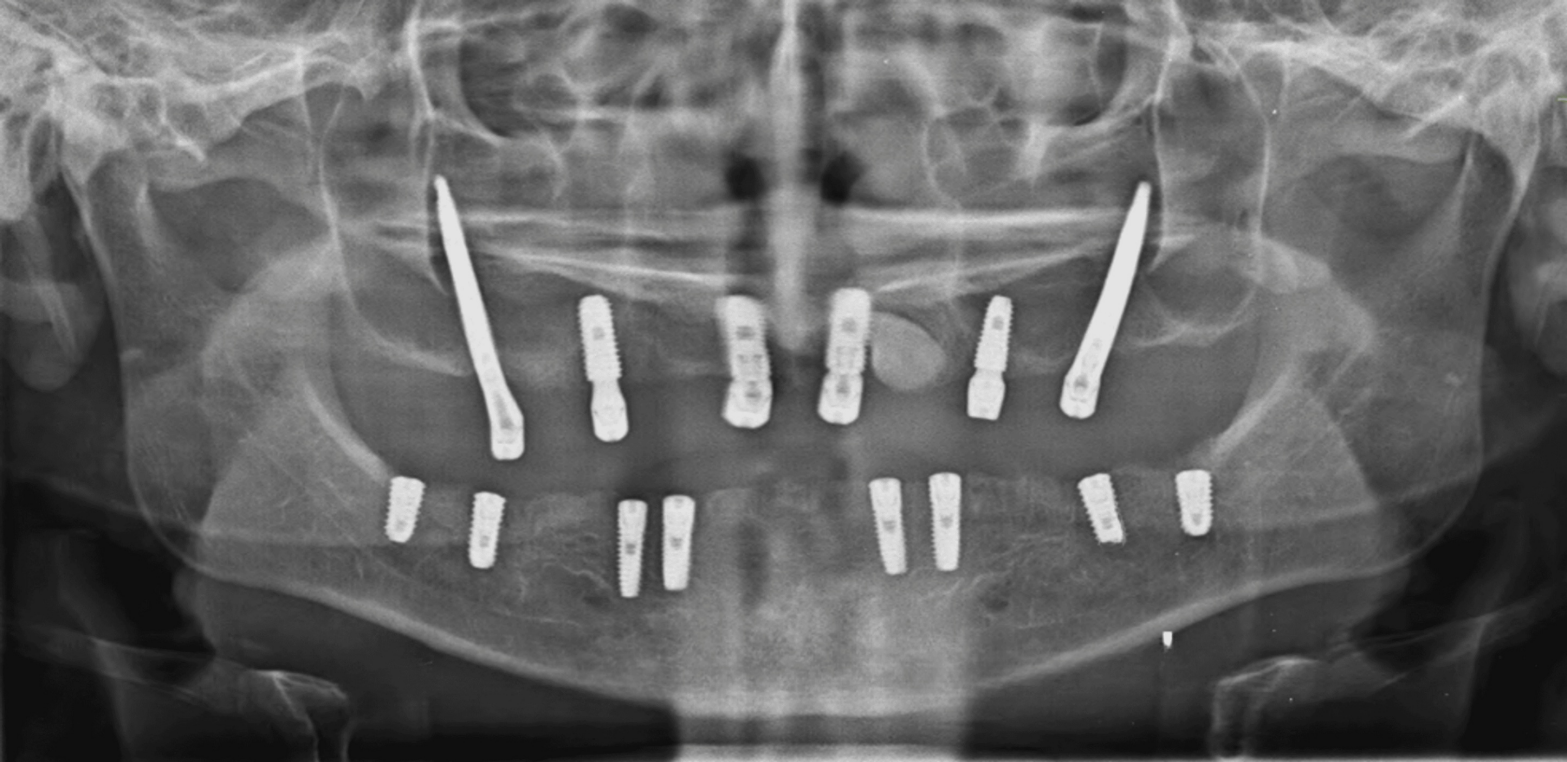
Postoperative OPG of the patient Image credit: Dr. Bhushan Mundada OPG: Orthopantomogram

The second stage of implant surgery was performed after four months. The post-operative X-rays showed good adaptation and osseointegration of all the implants with the bone. The patient was recalled after seven days for suture removal and prosthesis placement (Figures 7-8).

## Discussion

DD is a rare clinical entity that has an incidence of 1 in 100,000 live births [5]. It can be associated with other systemic conditions, such as skeletal sclerosis, Ehler-Danlos syndrome, and Brachio-Skeleto-Genital syndrome [6-8]. Differential diagnosis of DD includes other developmental anomalies of dentin formation, such as dentinogenetic imperfecta and regional odontodysplasia [9]. The exact pathophysiology of DD remains largely unknown. The difficulty in diagnosing DD can be due to its low incidence rate [5] and a lack of previous clinical experience, which may lead to a delay in reaching a correct diagnosis.

The management of DD is case-based, and the age of the patient seems to be a key influential factor. When diagnosed earlier, the treatment strategies are directed toward the retention of teeth for as long as possible. Akhil Jose et al. managed DD in 11-year-old patients symptomatically, using restorations for small caries and extraction of only the unrestorable teeth. They advocate preventive measures, such as oral prophylaxis, to control tooth mobility and intercept premature exfoliation. They suggest definitive management in the form of implant-supported prostheses after a patient is 18 years of age [2].

Implant-supported rehabilitation of patients with DD has been previously attempted [3,10-12]. The use of sinus lift procedures to counteract bone loss in the maxillary arch has been a time-tested method [3,10,11]. In contrast with this approach, we placed bilateral zygoma implants, which negated the need for sinus lift surgery. This greatly reduced the operating time, overall discomfort for the patient, and engagement in good cortical bone, with a minimum chance of resorption in the post-operative phase. To the best of our knowledge, this is the first case in which sinus lift surgery has not been performed in favor of zygoma implants in a patient with DD.

Due to reduced masticatory forces, bone resorption is frequently encountered in DD cases. Consequently, implant osseointegration and its success rate become unpredictable. To achieve optimal bony support, iliac bone grafting has been performed either in the lower arch or in both arches in the past. Additional measures, such as xenograft placement and platelet-rich plasma (PRP), have been tried to aid in the preservation of grafts and accelerate new bone formation [10,11]. We did not perform bone grafting, as the patient had adequate bone height, while the efficacy of PRP in accelerating bone formation is still debatable.

In the present case, all the implants showed good osteointegration, after four months of placement.

## Conclusions

The treatment of patients with DD depends on the age of the patient and the clinical severity of the disease. Adequate oral hygiene maintenance and dietary supplementation must be established and maintained for the retention of teeth to help children keep their natural teeth for as long as possible. In patients who have attained complete tooth eruption and skeletal maturity, oral rehabilitation must be planned to ensure proper quality of life and psychological well-being. Implant-supported rehabilitation preceded by sinus lift surgeries has been attempted in the past. The novel strategy of placing zygoma implants, which eliminates the need for sinus lift procedures, has been utilized for the initial instance in a patient with DD. This is a promising approach for the management of such patients and holds potential for further evaluation in the future.
